# Static and Fatigue Behavior Investigation of Artificial Notched Steel Reinforcement

**DOI:** 10.3390/ma10050532

**Published:** 2017-05-14

**Authors:** Yafei Ma, Qiang Wang, Zhongzhao Guo, Guodong Wang, Lei Wang, Jianren Zhang

**Affiliations:** School of Civil Engineering and Architecture, Changsha University of Science & Technology, Changsha 410114, China; fuyinjie1@sina.com (Q.W.); guozzhao@163.com (Z.G.); wangguodong6@outlook.com (G.W.); jianrenz@hotmail.com (J.Z.)

**Keywords:** fatigue testing, static testing, reinforcing bar, notch, stress concentration, fatigue life

## Abstract

Pitting corrosion is one of the most common forms of localized corrosion. Corrosion pit results in a stress concentration and fatigue cracks usually initiate and propagate from these corrosion pits. Aging structures may fracture when the fatigue crack reaches a critical size. This paper experimentally simulates the effects of pitting morphologies on the static and fatigue behavior of steel bars. Four artificial notch shapes are considered: radial ellipse, axial ellipse, triangle and length-variable triangle. Each shape notch includes six sizes to simulate a variety of pitting corrosion morphologies. The stress-strain curves of steel bars with different notch shape and depth are obtained based on static tensile testing, and the stress concentration coefficients for various conditions are determined. It was determined that the triangular notch has the highest stress concentration coefficient, followed by length-variable triangle, radial ellipse and axial ellipse shaped notches. Subsequently, the effects of notch depth and notch aspect ratios on the fatigue life under three stress levels are investigated by fatigue testing, and the equations for stress range-fatigue life-notch depth are obtained. Several conclusions are drawn based on the proposed study. The established relationships provide an experimental reference for evaluating the fatigue life of concrete bridges.

## 1. Introduction

Corrosion reduces the effective cross-sectional area of steel bars employed in concrete structures and leads to the deterioration of their mechanical properties. Corrosion of reinforcing bars is identified as one of the most predominant destructive factors of concrete bridges [1,2,3]. Corrosion is divided into general corrosion and pitting corrosion. For general corrosion, the corrosion rate over the entire reinforcing bar surface is relatively equal, and the cross-sectional area decreases uniformly. Pitting corrosion involves unequal corrosion rates over the surface of a reinforcing bar, which unevenly decreases the effective cross-sectional area, and thereby induces a stress concentration near the regions that suffer the greatest corrosion damage, i.e., the corrosion pits [4]. Previous experimental results showed that reinforcing bars primarily fracture at the corrosion pits, and pitting corrosion was considered to play a vital role in fatigue crack initiation and nucleation [5,6,7]. Some researchers generally assumed that the profiles of pitting corrosion points exhibit a semicircular or elliptical shape [8]. However, corrosion in practical processes is influenced by various factors, and large uncertainties exist [9]. Therefore, the geometries of corrosion pits are quite random. Currently, few studies focus on the mechanical properties of steel bars with different morphologies of pitting corrosion under static and fatigue loading conditions.

The mechanical properties of corroded rebars have been studied over the past few decades. The yield strength, the ultimate strength and the elongation of reinforcing bars were found to decrease with increasing corrosion level [10,11,12]. Some researchers such as Cairns [13] and Du [14] indicated that the reduction in the yield strength of corroded reinforcing bars presents an approximately linear relationship with respect to the corrosion loss by static tensile experiments and numerical simulations. Additionally, quantitative relationships for the yield strength and ultimate strength with respect to corrosion loss were obtained and some corresponding constitutive models were also proposed [15,16,17]. Most of these studies focused on the static loading effect on mechanical behavior of steel bars, and an average corrosion rate was typically applied without considering the influence of corrosion pits. However, the effects of pitting corrosion are not negligible, as has been discussed. Moreover, the bridges are subjected to an increasing stress range during service dependent on traffic volume, which increases the potential of fatigue damage as well.

The experimental results from the fatigue test performed by Apostolopoulos [18] and Li [19] indicated that the number of fatigue cycles of steel bars/cables decreases significantly with the increase of corrosion damage. Sun et al. [20] found that the stress-strain curve of corroded steel bars presented significant changes after fatigue and proposed a quantitative constitutive model based on the experimental results. However, very few studies considered the influence of pitting corrosion on the fatigue behavior of reinforcing bars. Fernandez et al. [21] identified that the corrosion pit depth has a much greater impact on the fatigue life than the pit length. A mechanical model was also presented to evaluate the effect of corrosion on fatigue behavior, where corrosion was implemented by an idealized pitted cross-section with double cross-section reduction [17]. In practical engineering, corrosion pits act more like notches because the pitting geometry is usually not the same as mathematically sharp cracks. Nakamura et al. [22] conducted a fatigue test using different shaped notches as the initial profiles of corrosion pits, which indicated that notch shape is a major factor reducing the fatigue strength of steel bars. Cerit et al. [23] established a three-dimensional model of corrosion pits using the finite element method by assuming the pit geometry has a semi-elliptical profile and by determining that pit aspect ratio is a major parameter affecting stress concentration factor. However, the precision of the simulation largely depends on the input parameters used in the model, and the pitting corrosion-induced stress concentration influence needs further verification. Ma et al. [24] proposed an empirical model of the stress concentration factor under different corrosion loss conditions, where the corrosion loss was represented by a mass loss of steel bars. The influence of the geometrical shape of the corrosion pit was not considered.

The present paper aims to investigate the effect of pitting corrosion morphologies on the static and fatigue behaviors of steel bars. The paper is organized as follows. First, a high-precision wire cutting technique is applied to fabricate samples with four notch shapes, seven notch lengths and six notch depths to simulate a variety of pitting corrosion morphologies on the surface of reinforcing bars. Next, the influences of the different notch shapes on the fatigue life are investigated using static axial tensile testing and fatigue testing. The stress concentration coefficients are determined for steel bars with different notch shapes and dimensions. Following this, the relationships between the fatigue life and the notch depth and the notch aspect ratio under different maximum stress levels are obtained. Equations for the stress range versus fatigue life as a function of notch depth are established. Finally, several conclusions are drawn based on the proposed study.

## 2. Experimental Program

### 2.1. Specimens Design

**Figure 1:**
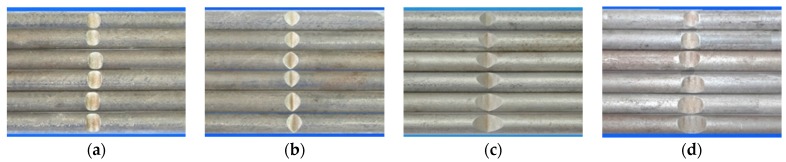
Specimens with different notch dimensions: (**a**) radial ellipse; (**b**) triangle; (**c**) length-variable triangle; (**d**) axial ellipse.

Hot-rolled plain round steel bars (HPB300) with 9 mm diameter were employed in the experiment. Each reinforcing bar is 40 cm in length. The average yield strength (*R_y_*) and ultimate strength (*R_m_*) are 446 MPa and 495 MPa, respectively. Four artificial notch shapes were considered: radial ellipse (RE), axial ellipse (AE), triangle (T), and length-variable triangle (VT). The notches with the same shape were designed to include six different dimensions, as shown in Figure 1. The notch depth *d* is in the range of 1.5–2.5 mm. The notch length *l* and the notch width *w* range from 7 to 12 mm and from 6.7 to 8.0 mm, respectively. The notches were located in the center of the test samples. The details can be found in Table 1.

**Figure 2:**
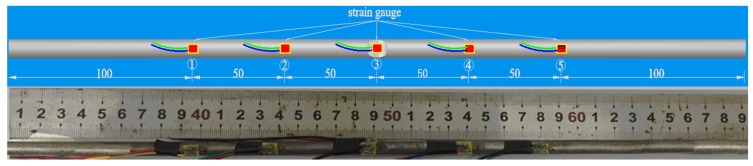
Arrangement of strain gauges on the notched specimen (unit: mm).

Four specimens were fabricated for each dimension, where three specimens were employed for fatigue testing and one specimen was employed for axial static tensile testing. Four virgin steel bars (three for the fatigue test and one for the static test) were used for comparison. The total number of specimens is one hundred. Each sample was equipped with five strain gauges. Figure 2 shows the arrangement of the strain gauges.

### 2.2. Loading Procedure

**Figure 3:**
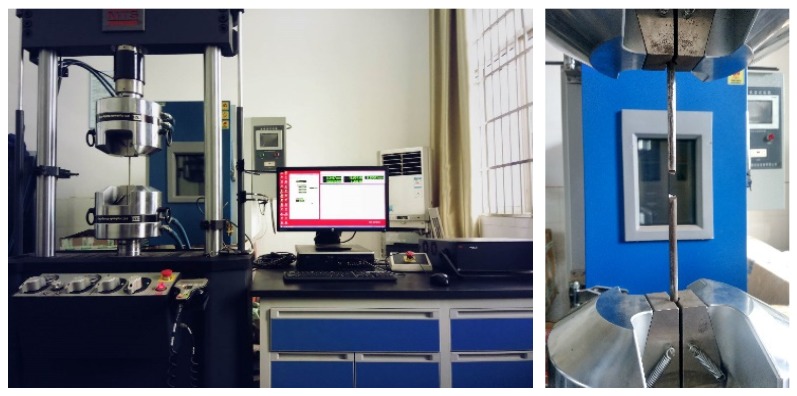
Test setup.

Both the static and fatigue tests were performed by the servo-hydraulic universal testing machine (MTS Landmark, Eden Prairie, MN, USA), as shown in Figure 3. The experiments were conducted in air at room temperature with reference to the GB/T228.1-2010 standard entitled Metallic materials-Tensile testing [25] and the British standard for steel for the reinforcement of concrete (BS4449-2005) [26]. The static loading process was controlled by deformation and the displacement rate was 5 mm/min. The applied load can also be monitored in all the cases by the MTS. Fatigue testing was conducted using a sinusoidal load with a loading frequency of 5 Hz and maximum stress levels of 178.4 MPa (0.4*R_m_*), 223 MPa (0.5*R_m_*), and 267.6 MPa (0.6*R_m_*), respectively. The stress is obtained using a nominal diameter of reinforcement. The stress ratio is 0.1.

## 3. Results and Discussion

### 3.1. Static Tensile Testing

The area loss of reinforcement results in a deterioration of material property. Apostolopoulos et al. [27] concluded that reinforcement area loss suffers a moderate reduction in tensile strength and a significant reduction in tensile ductility. Almusallam and Abdullah [28] identified a relationship between the cross-section loss and the strength of steel bars. In this section, the effects of various notches on the strength of steel bars are further investigated by static tensile testing. The stress-strain curves of specimens with different notch shapes and different notch depths are discussed. Following this, the notch-induced stress concentration coefficients are obtained based on the experimental observations.

Stress concentration occurs at the notch positions. The degree of stress concentration is generally represented by a theoretical stress concentration coefficient *K_tσ_*, which can be expressed as

(1) $$K_{t\sigma }=\frac{\sigma_{\max}}{\sigma_{\mathrm{nom}}}$$

where *σ_max_* is the maximum localized stress and *σ_nom_* is the nominal stress uniformly distributed across the cross-section of the reinforcing bar. In the current study, the stress concentration coefficient is obtained by moving from stress to the measured strain. The stress concentration coefficients are estimated from the ratio of maximum localized strain at notch root and average strain, i.e.,

(2) $$K_{t\sigma }=\frac{\varepsilon_{\max}}{\varepsilon_{nom}}$$

where *ɛ_max_* is the measured strain from gauge ③, and *ɛ_nom_* is the mean strain values measured from strain gauges ①, ②, ④, and ⑤ (average strain).

**Figure 4:**
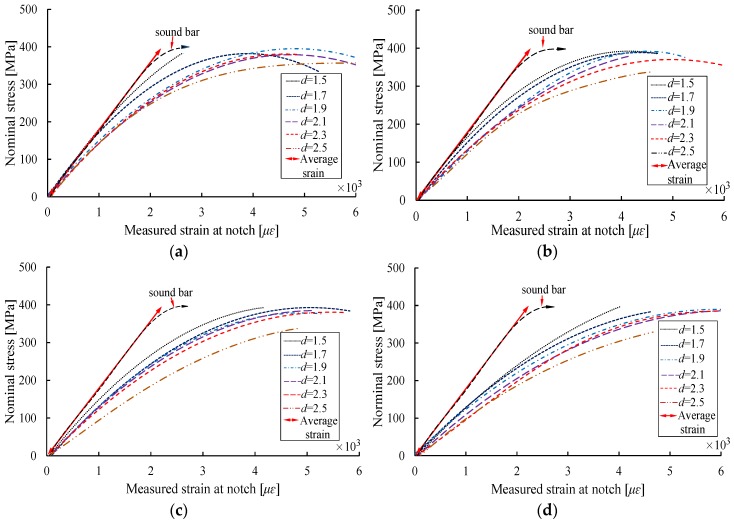
Stress-strain curves of specimens with different notch depths. (**a**) Axial ellipse; (**b**) radial ellipse; (**c**) length-variable triangle; (**d**) triangle.

Figure 4 shows the stress-strain curves for reinforcing bars with different notch shapes and different notch depths *d*, where the vertical axis represents the nominal stress value (ratio of the tensile force to the nominal cross-sectional area of the steel bar), and the horizontal axis corresponds to the measured strain corresponding to the strain gauge ③. As shown in Figure 4, the different shapes and depths of the notches results in stress concentration, such that the stress-strain curves for the various notches vary from each other. For a given notch shape, the strain at the notch exhibits an increasing trend with the increase of notch depth, and the growth rate of the strain at the notch increases with the increase of stress as compared with the average strain.

Figure 4a,b indicate that the strains at the AE and RE shaped notches are relatively close to the average strain at the initial loading process when the notch depth is less than 1.5 mm; as stress level increases, the differences between the strains at these two types of notches and the average strains gradually increase. For the VT and T shaped notches, the stress-strain curves vary significantly as a function of notch depth during the initial loading stage, as compared with the average strain.

**Figure 5:**
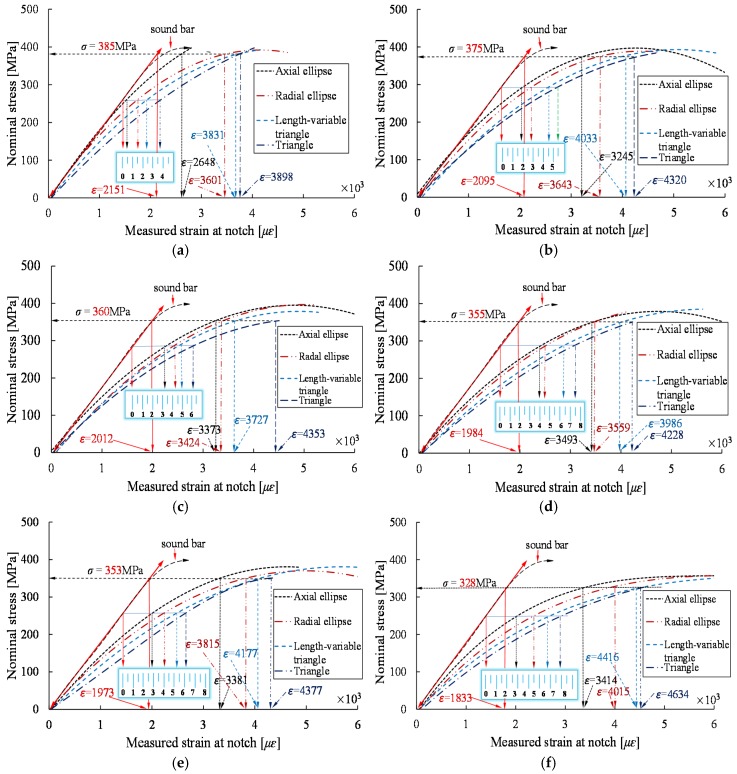
Stress-strain curves of samples with the same depths. (**a**) *d* = 1.5 mm; (**b**) *d* = 1.7 mm; (**c**) *d* = 1.9 mm; (**d**) *d* = 2.1 mm; (**e**) *d* = 2.3 mm; (**f**) *d* = 2.5 mm.

Figure 5 presents the stress-strain curves of steel bars with different notch shapes and the same notch depth. The small scale was used in the figure to quantitatively present the deviation degree between the strain at the notch and the average strain under the same stress level. As Figure 5 shows, for the same stress level, the T-shaped notch exhibits the largest strain, which indicates that the T-shaped notch has the largest stress concentration effect, followed by VT, RE and AE shaped notches. The nominal stresses of the T-shaped notches with the maximum depth *d* obtained at failure conditions are employed as the baseline stress values (the horizontal dashed arrow in Figure 5). The strains of the AE, RE, and VT shaped notches obtained at these stress values are employed as *ɛ_max_*, and the average values corresponding to these stress levels are shown as the *ɛ_nom_*.

**Figure 6:**
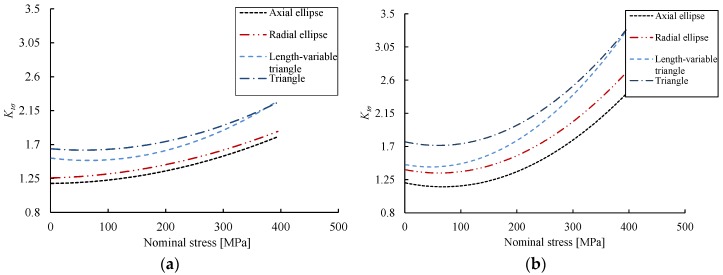
Evolution of stress concentration coefficient with respect to stress and notch shapes. (**a**) *d* = 2.1 mm; (**b**) *d* = 2.5 mm.

The values of *K_tσ_* for the reinforcing bar samples with different shaped notches and different notch depths are then calculated from Equation (2). Table 2 lists the calculation results of stress concentration coefficients. As indicated in Table 2, the T-shaped notch specimen has the maximum *K_tσ_* under a given notch depth, followed by VT, RE and AE. In addition, for illustration purposes, Figure 6 shows the evolution of *K_tσ_* up to failure with respect to different notch geometries at *d* = 2.1 mm and *d* = 2.5 mm for interested readers to understand the overall behavior of stress concentration, and a nonlinear increase is observed with the increase of stress levels.

### 3.2. Fatigue Testing

**Figure 7:**
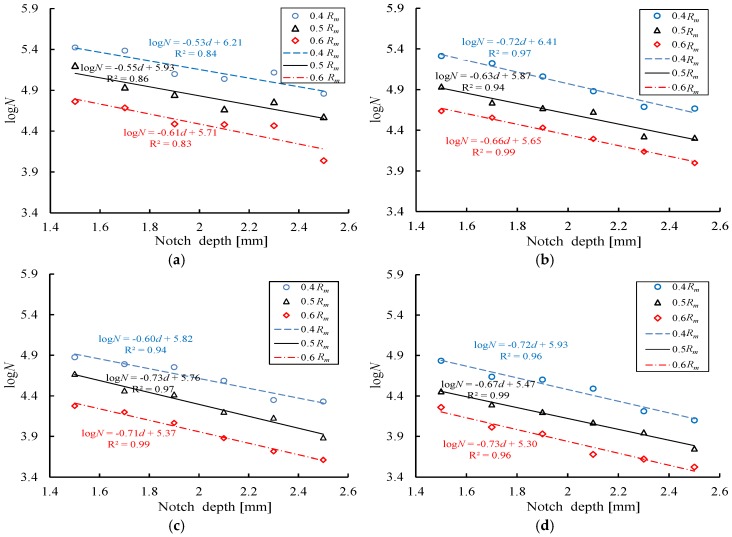
Comparison between the fatigue life of different notched steel bars. (**a**) Axial ellipse; (**b**) radial ellipse; (**c**) length-variable triangle; (**d**) triangle.

Table 3 shows the experimental results of the fatigue testing. Figure 7 presents the fatigue life of steel bars with the four notch shapes under three maximum stress levels, where the fatigue life is denoted here, and throughout, as the logarithm of the number of cycles to failure *N*. As Figure 7 shows, for a given stress level, the fatigue cycles of the reinforcing bars significantly decrease in a linear manner with increasing notch depth, and the slopes of decrease are approximately equivalent for each of the notch shapes. Therefore, a linear regression curve is used for the fitting process.

In addition, an increasing maximum stress level also shortens the fatigue life, and the decrease rate is associated with the notch geometry. In particular, the fatigue life for reinforcing bars with T-shaped notch has a larger decrease rate, while the specimens with AE-shaped notches exhibit a relatively smaller decrease rate. For example, the decrease rates of the fatigue life for the specimens with 1.5 mm T and AE shaped notches under 0.4*R_m_* are 93.3% and 74.2%, respectively. As the maximum stress level increases from 0.4*R_m_* to 0.6*R_m_*, the decrease rates of the above two-shaped notch specimens are 95.9% and 87.2%, respectively, which also indicates that the T-shaped notch has a more significant influence on the fatigue property than the AE-shaped notch.

**Figure 8:**
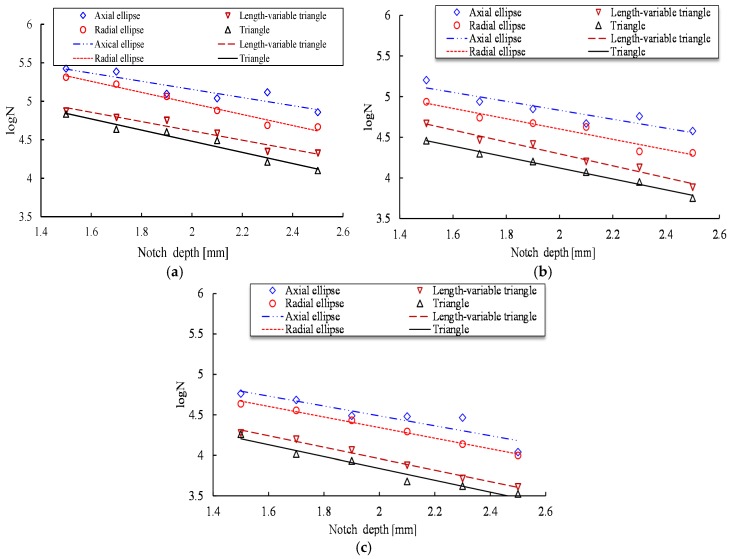
Fatigue life of steel bars under different maximum stress levels. (**a**) 0.4*R_m_*; (**b**) 0.5*R_m_*; (**c**) 0.6*R_m_*.

To quantitatively investigate the relationship between the four notch shape dimensions and the fatigue life of steel bars under various maximum stress levels, the data in Figure 7 is restructured in Figure 8 to present the fatigue life curves for the four types of notched specimens. As Figure 8 shows, for the sample with the same notch depth under the same stress level, the T-shaped notch and VT-shaped notch specimens have shorter fatigue life than those of the two other notched specimens due to different stress concentration factors. Based on the experimental results, it can be concluded that the previous assumptions of radial elliptical-shaped or axial elliptical-shaped corrosion pit may have a nonconservative result.

**Figure 9:**
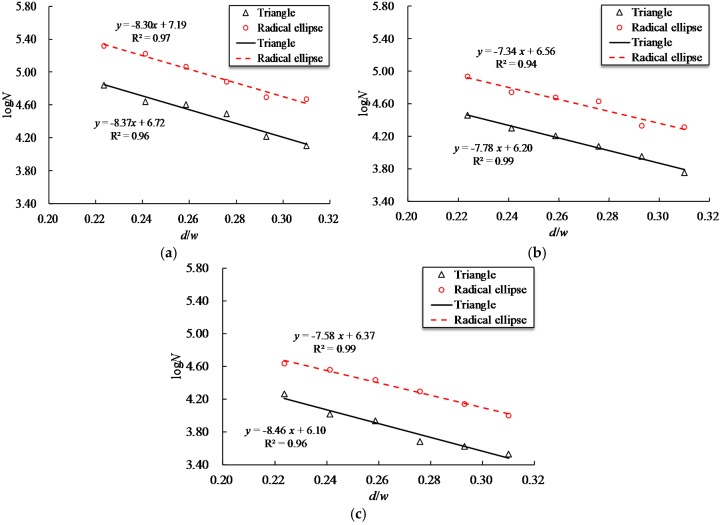
Relationship between fatigue life and *d*/*w* of triangular and radial elliptical notches at three stress levels. (**a**) 0.4*R_m_*; (**b**) 0.5*R_m_*; (**c**) 0.6*R_m_*.

The preceding results showed that the fatigue behavior of steel bars depends on the notch shapes and notch sizes. For this reason, a ratio of notch depth to notch width *w* (*d/w*) is selected to further evaluate the joint influence of these two factors on the fatigue life of the reinforcing bar. Figure 9 indicates the fatigue life with respect to the aspect ratio *d/w* for steel bars with T and RE shaped notches at the three maximum stress levels. As Figure 9 shows, the decrease rate in the fatigue life for reinforcing bar with a T-shaped notch is faster than that with an RE-shaped notch under equivalent maximum stress level. This indicates that reinforcing bars with a T-shaped notch are more sensitive to *d/w* than those with an RE-shaped notch. The least square method was employed to fit the logN versus *d/w* data for RE and T shaped notches, which provides the following expressions.

(3) $$logN_{RE}=\left\{ \begin{array}{l} -\frac{4.15}{\sqrt{9/d_{0.4R_{m}}-1}}+7.19\\ -\frac{3.67}{\sqrt{9/d_{0.5R_{m}}-1}}+6.56\\ -\frac{3.79}{\sqrt{9/d_{0.6R_{m}}-1}}+6.16 \end{array}\right.$$

(4) $$logN_{T}=\left\{ \begin{array}{l} -\frac{4.19}{\sqrt{9/d_{0.4R_{m}}-1}}+6.72\\ -\frac{3.89}{\sqrt{9/d_{0.5R_{m}}-1}}+6.20\\ -\frac{4.23}{\sqrt{9/d_{0.6R_{m}}-1}}+6.10 \end{array}\right.$$

where *N_RE_* and *N_T_* are the fatigue life of reinforcing bars with RE and T shaped notches, respectively, and the subscript on *d* represents the maximum stress level.

**Figure 10:**
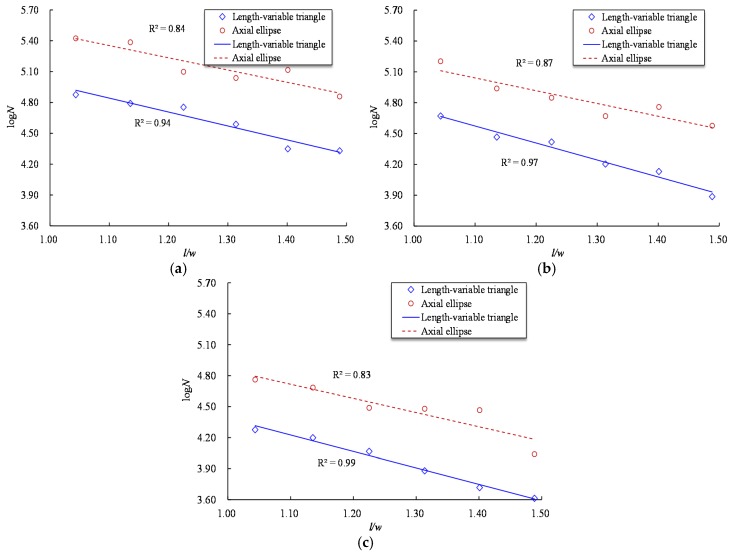
Relationship between fatigue life and *l*/*w* of length-variable triangular and axial elliptical shaped notches at three stress levels. (**a**) 0.4*R_m_*; (**b**) 0.5*R_m_*; (**c**) 0.6*R_m_*.

The fatigue life of a notched reinforcing bar is also related to the notch length *l*. A comparison for the fatigue life curves of steel bars with T and VT shaped notches indicates that the VT notched specimen has a longer fatigue life for a given notch depth and stress level, which is consistent with the observed results from the AE and the RE shaped notch specimens, i.e., a longer fatigue life is related to a longer notch length. Figure 10 presents the fatigue life of reinforcing bars with VT and AE shaped notches with respect to the ratio *l/w*. As Figure 10 shows, the logN exhibits an inverse linear relationship with respect to the *l/w* for the specimens with VT and AE shaped notches, and the difference in fatigue life gradually increases with the increase of *l/w*. The relationships between the logN and *l/w* for VT and AE-shaped notches can be expressed as

(5) $$logN_{VT}=\left\{ \begin{array}{l} -1.19r_{0.4R_{m}}+6.67\\ -1.24r_{0.5R_{m}}+6.41\\ -1.37r_{0.6R_{m}}+6.23 \end{array}\right.$$

(6) $$logN_{AE}=\left\{ \begin{array}{l} -1.35r_{0.4R_{m}}+6.33\\ -1.65r_{0.5R_{m}}+6.39\\ -1.59r_{0.6R_{m}}+5.98 \end{array}\right.$$

where *N_VT_* and *N_AE_* represent the fatigue life of steel bars with VT and AE-shaped notches, respectively, *r* = *l*/*w*, and the subscript on *r* represents the maximum stress level.

Generally, fatigue test results are presented in the form of the relationship between fatigue life of specimens and the stress range (*S*). A linear relationship can be expressed mathematically as

(7) logN+mlogS=logA

where *m* and *A* are both constants of the material.

**Figure 11:**
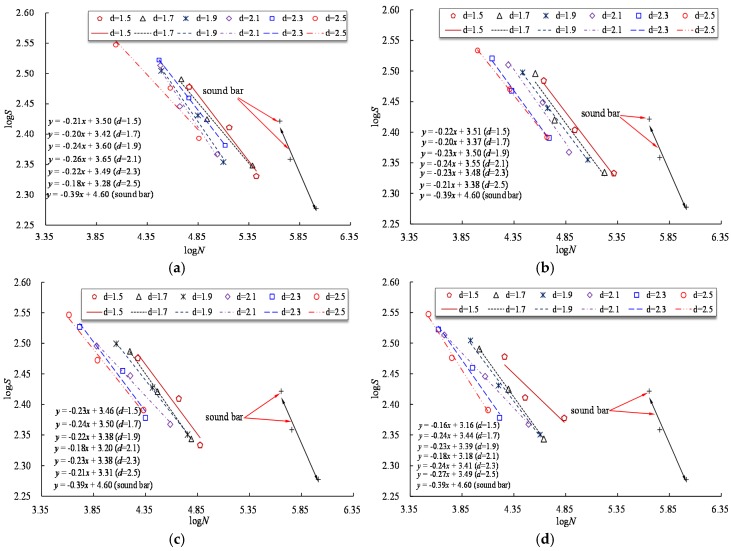
Log*S* versus log*N* curves for specimens with different notch shapes. (**a**) Axial ellipse; (**b**) radial ellipse; (**c**) length-variable triangle; (**d**) triangle.

Figure 11 presents the log*S* versus log*N* curves for reinforcing bars with different notch shapes and different notch sizes. The notch decreases the effective area of a reinforcing bar, and the stress range is calculated here using the actual cross-sectional area. As Figure 11 shows, a linear relationship of the log*S* versus log*N* curve is observed for the specimen with the same notch shape but different notch depth. Figure 11 also indicates that log*N* decreases rapidly with increasing notch depth for a fixed log*S*. In practical engineering, the fatigue loading experienced by in service bridges continuously increases with the growth of traffic volume. The increasing stress range of steel bars increases the fatigue failure possibility of aging bridge structures, which should be highly concerning for the bridge management department.

## 4. Conclusions

The effect of simulated pitting corrosion morphologies on the static and fatigue behaviors of steel bars were investigated in this paper. Based on the experimental results, the following conclusions can be drawn.

- The experimental static axial tensile testing and fatigue testing results show that the T-shaped notch has the highest stress concentration coefficient, followed by VT, RE, and AE shaped notches. The degree of stress concentration at the notch increases with increasing applied stress and increasing notch depth.
- The fatigue life of reinforcing bars significantly decreases as notch depth increases. Reinforcing bars with T-shaped notches are more sensitive to the aspect ratio *d*/*w* than those with RE-shaped notches. The difference in fatigue life for the two types of notches gradually increase with the increase of *l*/*w*.
- Fatigue curve equations for stress range-fatigue life-notch depth are established by linear regression, which provides an experimental basis for the fatigue life assessment of aging concrete bridges.
- The stress concentration coefficient exhibits an overall increasing trend with the increase of notch depth, while different uncertainty behavior is observed for this trend (due to intrinsic properties of the material and processing technique) and probabilistic inference may be a rational way to describe these uncertainties.

The proposed study uses artificial notches to simulate the effect of corrosion pit on the static and fatigue behaviors of reinforcement, which may differ from the natural corrosion process. This study only considers the influence of a single notch type on the mechanical behavior of steel bars. The correlation between various notch types and their effects requires further investigation. Future work to perform experimental study or collect actual data for validation is required.

## Figures and Tables

**Table 1:** Design of notch shape and notch size.

Specimen No.	Notch Shape and Size (Unit: mm)			Specimen No.	Notch Shape and Size (Unit: mm)		
	Side View	Top View	Photos of Notch		Side View	Top View	Photos of Notch
RE5−6.7−1.5	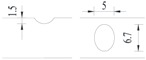		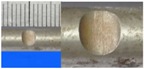	AE7−6.7−1.5	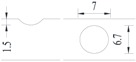		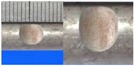
RE5−7.0−1.7	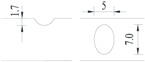		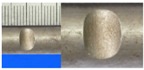	AE8−7.0−1.7	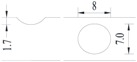		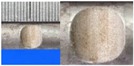
RE5−7.3−1.9	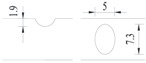		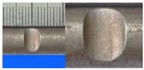	AE9−7.3−1.9	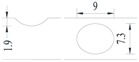		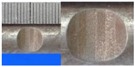
RE5−7.6−2.1	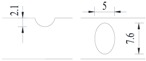		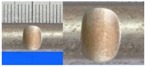	AE10−7.6−2.1	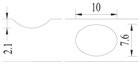		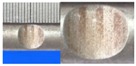
RE5−7.9−2.3	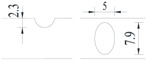		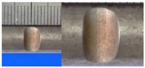	AE11−7.9−2.3	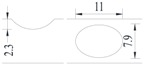		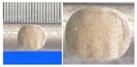
RE5−8.0−2.5	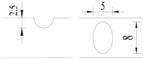		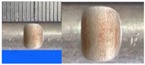	AE12−8.0−2.5	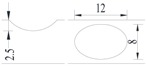		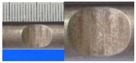
T5−6.7−1.5	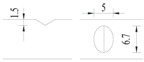		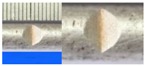	VT7−6.7−1.5	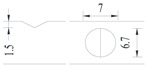		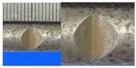
T5−7.0−1.7	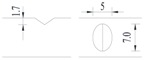		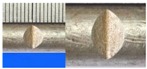	VT8−7.0−1.7	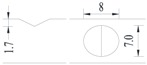		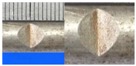
T5−7.3−1.9	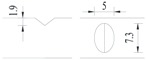		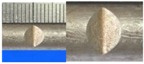	VT9−7.3−1.9	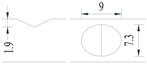		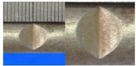
T5−7.6−2.1	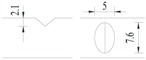		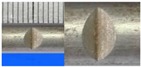	VT10−7.6−2.1	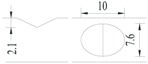		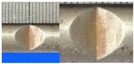
T5−7.9−2.3	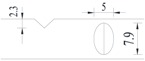		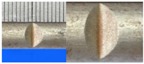	VT11−7.9−2.3	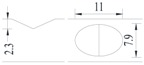		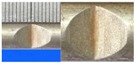
T5−8.0−2.5	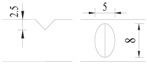		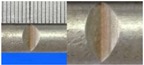	VT12−8.0−2.5	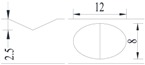		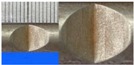

Note: The specimen number in the first column corresponds to the notch size.

**Table 2:** Stress concentration coefficients for different notch shapes and notch depths.

Notch Depth (mm)	Nominal Stress (MPa)	Measured Strain at the Notch (*με*)				Average Strain (*με*)	Stress Concentration Coefficient			
		AE	RE	VT	T		AE	RE	VT	T
1.5	385	2648	3601	3831	3898	2151	1.231	1.674	1.781	1.812
1.7	375	3245	3643	4033	4320	2095	1.549	1.739	1.925	2.062
1.9	360	3373	3424	3727	4353	2012	1.676	1.702	1.852	2.164
2.1	355	3493	3559	3986	4228	1984	1.761	1.794	2.009	2.131
2.3	353	3381	3815	4177	4377	1973	1.714	1.934	2.117	2.218
2.5	328	3414	4015	4416	4634	1833	1.863	2.190	2.409	2.528

**Table 3:** Experimental results of fatigue testing.

Specimen No.	Stress Levels	Minimum Area/mm^2^	Stress Range/MPa	Fatigue Life/Cycle	Specimen No.	Stress Levels	Minimum Area/mm^2^	Stress Range/MPa	Fatigue Life/Cycle
0−0−0	0.4*R_m_*	63.5	189.4	1,028,469	VT12−8.0−2.5	0.6*R_m_*	47.8	352.1	4100
0−0−0	0.5*R_m_*	63.2	228.3	571,120	RE5−6.7−1.5	0.4*R_m_*	55.8	217.7	205,791
0−0−0	0.6*R_m_*	63.8	264.0	451,763	RE5−6.7−1.5	0.5*R_m_*	56.9	253.5	86,571
T5−6.7−1.5	0.4*R_m_*	56.2	213.8	68,466	RE5−6.7−1.5	0.6*R_m_*	55.2	305.2	43,342
T5−6.7−1.5	0.5*R_m_*	56.4	255.6	28,745	RE5−7.0−1.7	0.4*R_m_*	55.7	215.4	167,112
T5−6.7−1.5	0.6*R_m_*	55.3	304.1	18,225	RE5−7.0−1.7	0.5*R_m_*	54.9	263.6	55,132
T5−7.0−1.7	0.4*R_m_*	55.0	218.6	43,459	RE5−7.0−1.7	0.6*R_m_*	53.7	313.8	35,904
T5−7.0−1.7	0.5*R_m_*	55.3	260.7	19,848	RE5−7.3−1.9	0.4*R_m_*	53.1	228.0	115,350
T5−7.0−1.7	0.6*R_m_*	54.4	309.4	10,380	RE5−7.3−1.9	0.5*R_m_*	52.5	275.0	47,168
T5−7.3−1.9	0.4*R_m_*	53.4	225.0	40,055	RE5−7.3−1.9	0.6*R_m_*	53.5	313.5	27,079
T5−7.3−1.9	0.5*R_m_*	53.4	270.4	15,928	RE5−7.6−2.1	0.4*R_m_*	51.6	233.0	75,904
T5−7.3−1.9	0.6*R_m_*	53.4	315.4	8,545	RE5−7.6−2.1	0.5*R_m_*	51.3	283.7	42,406
T5−7.6−2.1	0.4*R_m_*	51.9	231.9	30,998	RE5−7.6−2.1	0.6*R_m_*	52.0	322.9	19,701
T5−7.6−2.1	0.5*R_m_*	51.5	280.0	11,813	RE5−7.9−2.3	0.4*R_m_*	48.9	246.0	48,764
T5−7.6−2.1	0.6*R_m_*	51.4	327.2	4,775	RE5−7.9−2.3	0.5*R_m_*	49.1	295.3	21,215
T5−7.9−2.3	0.4*R_m_*	48.7	246.7	16,282	RE5−7.9−2.3	0.6*R_m_*	50.8	333.7	13,725
T5−7.9−2.3	0.5*R_m_*	49.7	290.3	8928	RE 5−8.0−2.5	0.4*R_m_*	48.8	246.5	46,466
T5−7.9−2.3	0.6*R_m_*	50.1	323.1	4188	RE 5−8.0−2.5	0.5*R_m_*	48.9	296.9	20,343
T5−8.0−2.5	0.4*R_m_*	49.0	245.6	12,606	RE 5−8.0−2.5	0.6*R_m_*	49.3	341.7	9951
T5−8.0−2.5	0.5*R_m_*	49.2	293.1	5643	AE7−6.7−1.5	0.4*R_m_*	56.1	214.3	265,329
T5−8.0−2.5	0.6*R_m_*	49.4	341.1	3354	AE7−6.7−1.5	0.5*R_m_*	56.0	257.8	159,663
T5−6.7−1.5	0.4*R_m_*	55.8	215.6	75,066	AE7−6.7−1.5	0.6*R_m_*	56.0	300.8	57,889
VT7−6.7−1.5	0.5*R_m_*	56.2	256.9	46,853	AE8−7.0−1.7	0.4*R_m_*	53.	223.0	243,127
VT7−6.7−1.5	0.6*R_m_*	54.2	299.3	18,886	AE8−7.0−1.7	0.5*R_m_*	54.3	268.9	86,707
VT8−7.0−1.7	0.4*R_m_*	54.5	220.5	61,772	AE8−7.0−1.7	0.6*R_m_*	54.4	311.0	48,373
VT8−7.0−1.7	0.5*R_m_*	54.7	263.6	29,207	AE9−7.3−1.9	0.4*R_m_*	53.2	225.9	125,429
VT8−7.0−1.7	0.6*R_m_*	54.9	306.8	15,816	AE9−7.3−1.9	0.5*R_m_*	53.5	275.3	70,545
VT9−7.3−1.9	0.4*R_m_*	53.6	224.2	56,824	AE9−7.3−1.9	0.6*R_m_*	52.7	323.4	30,753
VT9−7.3−1.9	0.5*R_m_*	53.9	267.7	26,166	AE10−7.6-2.1	0.4*R_m_*	51.7	232.7	109,326
VT9−7.3−1.9	0.6*R_m_*	53.3	315.7	11,676	AE10−7.6−2.1	0.5*R_m_*	51.7	279.2	46,792
VT10−7.6-2.1	0.4*R_m_*	51.6	233.1	38,688	AE10−7.6−2.1	0.6*R_m_*	51.6	316.7	30,201
VT10−7.6−2.1	0.5*R_m_*	51.6	279.9	15,985	AE11−7.9−2.3	0.4*R_m_*	49.9	240.8	130,749
VT10−7.6−2.1	0.6*R_m_*	53.8	313.1	7572	AE11−7.9−2.3	0.5*R_m_*	50.0	290.0	57,385
VT11−7.9−2.3	0.4*R_m_*	50.4	238.8	22,353	AE11−7.9−2.3	0.6*R_m_*	50.6	332.9	29,229
VT11−7.9−2.3	0.5*R_m_*	50.6	285.0	13,508	AE12−8.0−2.5	0.4*R_m_*	48.6	250.1	72,154
VT11−7.9−2.3	0.6*R_m_*	50.0	336.4	5209	AE12−8.0−2.5	0.5*R_m_*	48.2	300.3	37,698
VT12−8.0−2.5	0.4*R_m_*	48.9	246.0	21,388	AE12−8.0−2.5	0.6*R_m_*	47.7	352.7	10,946
VT12−8.0−2.5	0.5*R_m_*	48.6	296.8	7706					

Note: the minimum area corresponds to the notch center section and can be calculated by geometric analysis with a known notch depth.
